# Failing beta-cell adaptation in South Asian families with a high risk of type 2 diabetes

**DOI:** 10.1007/s00592-014-0588-9

**Published:** 2014-05-05

**Authors:** Sjaam Jainandunsing, Behiye Özcan, Trinet Rietveld, Joram N. I. van Miert, Aaron J. Isaacs, Janneke G. Langendonk, Felix W. M. de Rooij, Eric J. G. Sijbrands

**Affiliations:** 1 Department of Internal Medicine, Erasmus MC - University Medical Center Rotterdam, Room Bd-299, PO Box 2040, 3000 CA Rotterdam, The Netherlands; 2 Department of Epidemiology, Erasmus MC - University Medical Center Rotterdam, Rotterdam, The Netherlands

**Keywords:** Type 2 diabetes, OGTT: insulin, Glucose, South Asian

## Abstract

We performed an extended oral glucose tolerance test (OGTT) to investigate the relationship between early and late beta-cell response and type 2 diabetes (T2D) in families of South Asian origin and indigenous Dutch, burdened by T2D. Based on the OGTT, 22 individuals were normoglycemic, 12 glucose intolerant and 23 had T2D in the South Asian families; these numbers were 34, 12 and 18 in the Caucasian families, respectively. The OGTT had 11 blood samplings in 3.5 h for glucose, insulin and C-peptide measurements. Through early and late insulin secretion rate (ISR), the above basal glucose area-under-the-curve after glucose load (glucose disposal) and insulin sensitivity index (ISI), we obtained early and late disposition indices (DI). South Asians on average had lower ISI than Caucasians (3.8 ± 2.9 vs. 6.5 ± 4.7, respectively, *P* < 0.001), with rapid decline of their early and late DI between normal glucose tolerance versus impaired fasting glucose/impaired glucose tolerance (late DI; *P* < 0.0001). Adjusted for ISI, age, gender and waist-to-hip ratio, early ISR was significantly associated with glucose disposal in South Asians (*β* = 0.55[0.186; 0.920]), but not in Caucasians (*β* = 0.09[−0.257; 0.441]). Similarly, early ISR was strongly associated with late ISR (*β* = 0.71[0.291; 1.123]; *R* ^2^ = 45.5 %) in South Asians, but not in Caucasians (*β* = 0.27[−0.035; 0.576]; *R* ^2^ = 17.4 %), with significant interaction between ethnicity and early ISR (*β* = 0.341[0.018; 0.664]). Ordinal regression analyses confirmed that all South Asian OGTT subgroups were homogenously resistant to insulin and solely predicted by early ISR (*β* = −0.782[−1.922; 0.359], *β* = −0.020[−0.037; −0.002], respectively), while in Caucasian families both ISI and early ISR were related to glucose tolerance state (*β* = −0.603[−1.105; −0.101], *β* = −0.066[−0.105; −0.027], respectively). In South Asian individuals, rapid beta-cell deterioration might occur under insulin resistant conditions. As their early insulin response correlates strongly with both glucose disposal and late insulin response, alterations in beta-cell dynamics may give an explanation to their extreme early onset of T2D, although larger prospective studies are required.

**Electronic supplementary material:**

The online version of this article (doi:10.1007/s00592-014-0588-9) contains supplementary material, which is available to authorized users.

## Introduction

Dutch citizens of South Asian origin have a nearly fivefold higher prevalence of type 2 diabetes (T2D) than the indigenous Dutch population (further described as Caucasian) [1, 2]. The increased susceptibility to T2D is also evident from the early onset of the disorder at relatively low body mass and the remarkably high incidence of cardiovascular and microvascular damage among the South Asians [2, 3]. A number of factors have been proposed to account for this strikingly high risk in South Asians, including a high prevalence of metabolic syndrome, impaired maternal lipid profile conditions, low birth weight causing central obesity later in life, dysfunction of adipocytes, as well as educational, social and economic inequalities [4–14]. These factors all enhance insulin resistance and promote hyperinsulinemia [14, 15]. In addition, T2D is characterized by beta-cell dysfunction. Genetic loci predisposing individuals to T2D affect both beta-cell function and insulin action [16, 17].

The ancestors of South Asian families in the Netherlands moved from a circumscribed region in India to Surinam. During the past 150 years, these South Asian families lived largely in genetic isolation before arriving in the Netherlands. The conservation of susceptibility loci may have contributed to the strong aggregation of T2D in these families. We hypothesized that, in addition to severe resistance to insulin, these South Asian families are also predisposed to develop beta-cell dysfunction. Therefore, we investigated beta-cell function and insulin sensitivity simultaneously in South Asian and Caucasian patients with T2D and first-degree relatives. In effect, we assessed the contribution to the risk of T2D of changes in early and late insulin secretion rates (ISR) and insulin sensitivity during an extended oral glucose tolerance test (OGTT) with insulin and C-peptide measurements.

## Methods

### Subjects

**Scheme 1:**
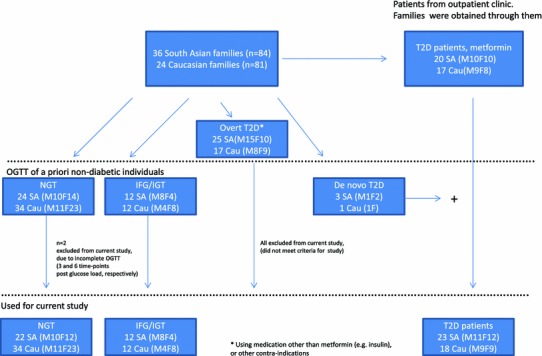
Inclusion flow chart of individuals from South Asian and Caucasian families

The study was conducted during a time period between August 2007 and January 2011. Patients with T2D and first-degree relatives without T2D were recruited from 36 South Asian families and 24 Caucasian families (Scheme 1). Power calculation was performed with Quanto version 1.0 [31] and was based on differences in early phase ISR (described further on in Methods section) between healthy South Asian and Caucasian performed in a pilot phase of the study among, with alpha 0.05 and power 80 %. All probands were attending the outpatient clinic of the Department of Internal Medicine of the Erasmus Medical Center in Rotterdam. T2D was diagnosed according to World Health Organization (WHO) criteria [18]: plasma glucose level ≥7.0 mmol/L in a fasting state and/or ≥11.1 mmol/L in a non-fasting state. Inclusion criteria for probands were age of 18 years or older and T2D in at least one sibling. Both parents of the South Asian probands were of South Asian origin, and Caucasian probands were born in the Netherlands with both parents of Caucasian Dutch origin. Exclusion criteria were insulin-dependent diabetes mellitus, using medication other than metformin, a history of pancreatitis, insulinoma or other reasons that made participation impossible. Written informed consent was obtained from all participants. The study protocol was approved by the Erasmus Medical Center Medical Ethics Review Board.

### Physical examination

Body height and weight were measured to the nearest 0.1 cm and 0.1 kg for the determination of body mass index (BMI). Waist circumference was measured in cm halfway between the lowest rib and the iliac crest, the maximum circumference of the hips was measured in the standing position in cm, and, from these measurements, the waist-to-hip ratio (W/H) was calculated. Systolic and diastolic blood pressures were measured with an electronic blood pressure monitor (Datascope Accutorr Plus Inc., Montvale, NJ) after five minutes rest in the sitting position.

### Oral glucose tolerance test (OGTT)

Glucose, 75 g dissolved in 200 ml H_2_O, was administered orally after a 10-h overnight fast. Venous blood was drawn via an intravenous canula, 60 and 15 min before the glucose load and 15, 30, 45, 60, 90, 120, 150, 180 and 210 min after glucose loading. WHO criteria based on OGTT were used to define family members with normal glucose tolerance (NGT), impaired fasting glucose/impaired glucose tolerance (IFG/IGT) and T2D [18].

### Assays

Plasma glucose was measured by a hexokinase-based method (Gluco-quant; Roche Diagnostics, Mannheim, Germany). Plasma insulin and C-peptide were measured separately by a competitive chemiluminescent immunoassay, supplied by Euro/DPC (Diagnostic Product Corporation, Los Angeles, CA). The assay was performed on a DPC Immulite 2000 analyzer (Euro/DPC) according to the manufacturer’s recommended protocol.

### Calculation of indices

#### Beta-cell function indices

For the assessment of early, late and overall beta-cell function, we calculated incremental ISR area-under-the curves (AUCs); ISR t0–30 and ISR t60–210 and ISR t0–210, respectively, based on plasma C-peptide concentrations with ISEC software [19]. The ISR reflects the prehepatic secretion rate, as C-peptide has negligible hepatic clearance. Hereafter, we investigated early, late and overall beta-cell function in relation to glucose concentrations and insulin sensitivity to obtain early, late, and overall disposition indices (DI), respectively.

#### Insulin sensitivity

The insulin sensitivity index (ISI) was determined according to [20, 21]

$$ {\text{ISI}} = 10.000/(G_{0} \times I_{0} \times {\text{mean}}G_{{0{-} 30{-} 60{-} 90{-} 1 20}} \times {\text{mean}}I_{{0{-} 30{-} 60{-} 90{-} 1 20}} )^{ 1/ 2} . $$

#### Early, late and overall Disposition Indices

Early, late and overall DI were calculated as follows: ISR t0–30/glucose disposal t0–30 × ISI, ISR t60–210/glucose disposal t60–210 × ISI and ISR t0–210/glucose disposal t0–210 × ISI, respectively [22]. In addition, we calculated the ratio of late phase DI to early phase DI, based on earlier observations marking their relationship [23].

To improve comparison with previous studies, we also added a large number of classical indices to online supplemental Table 1 and supplemental Figure 1. All formulae are described below the online supplemental Table 1. All OGTT indices were derived from insulin and C-peptide concentrations in pmol/L and glucose concentrations in mmol/L, with the exception that insulin concentrations were converted to μU/mL for the calculation of HOMA and ISI and, subsequently, DIs were both calculated with glucose in mg/dL. All AUCs were calculated according to the trapezoid method, and incremental AUCs were calculated by subtracting basal values from total calculated AUC values between given time points [24].

**Table 1:** Clinical characteristics of the NGT, IGT and/or IFG and T2D subgroups

	NGT SA	NGT Cau	IFG/IGT SA	IFG/IGT Cau	T2D SA	T2D Cau
*n*	22	34	12	12	23	18
Sex(male/female), *n* %(male)	10/12 (45.5)	11/23 (32.4)	8/4 (66.7)	4/8 (33.3)	11/12 (47.8)	9/9 (50.0)
Age (years)	39.6 ± 11.6^¶^	38.9 ± 9.4^‡^	46.3 ± 8.8	44.5 ± 11.4^‡^	52.3 ± 8.8^‡§^	63.2 ± 7.6*^†¶^
Weight (kg)	78.7 ± 13.8	81.1 ± 15.7	78.7 ± 14.5	94.1 ± 30.4	74.4 ± 12.4	90.5 ± 15.0^¶^
Length (cm)	1.69 ± 0.1	1.75 ± 0.1	1.67 ± 0.1	1.75 ± 0.1	1.61 ± 0.1^‡^	1.76 ± 0.1^¶^
BMI (kg/m^2^)	27.6 ± 4.1	26.3 ± 4.1	27.9 ± 2.9	30.4 ± 8.5	28.6 ± 4.1	29.3 ± 4.9
Waist (cm)	94 ± 10	91 ± 15^‡^	98 ± 13	105 ± 20	97 ± 11	105 ± 14*
Hip (cm)	105 ± 5	108 ± 8	103 ± 6	116 ± 20	104 ± 9	112 ± 10
W/H	0.90 ± 0.07	0.84 ± 0.09^‡^	0.95 ± 0.10	0.90 ± 0.07	0.93 ± 0.08	0.94 ± 0.08*
RR systolic (mmHg)	122.6 ± 14.1	123.2 ± 12.5	125.9 ± 15.0	129.6 ± 20.2	132.4 ± 15.1	135.6 ± 13.0
RR diastolic (mmHg)	77.0 ± 9.7	76.3 ± 9.1	85.0 ± 10.8	79.4 ± 10.3	80.4 ± 8.4	83.4 ± 12.2
Smoking, *n* %^D^	2 (11.8)	13 (40.6)	(5) 55.6	(5) 50.0	8 (50.0)	5 (50.0)
Antihypertensive, *n* %	2 (9.1)	0^‡^	2 (16.7)	3 (25.0)	9 (39.1)	9 (50.0)*
Lipid treatment, *n* %	3 (13.6)	1 (2.9)^‡^	3 (25.0)	0^‡^	11 (47.8)	11 (61.1)*^†^
Macrovascular history, *n* %	4.5	3.0	16.7	0	8.7	12.5*
Microvascular history, *n* %					13.0^B^	25.0^B^
Metformin usage					20(87 %)	17(94.4 %)
Age of diagnosis					44.3 ± 7.3^A,B^	56.1 ± 7.2^A,B^
Period of having T2D					9.9 ± 7.3^A,C^	8.5 ± 8.4^A,C^
Fasting glucose (mmol/L)	5.3 ± 0.4^¶^	5.2 ± 0.3^†‡^	6.0 ± 0.5^¶^	6.0 ± 0.6*^‡^	7.2 ± 1.1^§||^	8.0 ± 1.1*^†^
120 min glucose (mmol/L)	5.4 ± 1.1^¶^	5.5 ± 1.1^‡^	7.8 ± 1.1^¶^	7.7 ± 2.3*^‡^	12.5 ± 4.4^§||^	14.1 ± 3.6*^†^
ISI	5.0 ± 3.9	8.2 ± 5.1^‡^	3.2 ± 1.8	5.0 ± 3.2	2.9 ± 1.6	4.2 ± 3.3*
ISR t0–210	1,647 ± 852	1,153 ± 385	1,873 ± 862	1,369 ± 561	1,645 ± 513^‡^	1,060 ± 478
ISR t0–30	297 ± 122*^¶^	208 ± 82^‡^	254 ± 158^¶^	166 ± 69	116 ± 64^§||^	124 ± 75
ISR t60–210	921 ± 632	595 ± 296	1,217 ± 699	900 ± 524	1,225 ± 430^‡^	744 ± 360
Glucose disposal t0–210	169 ± 95^¶^	211 ± 126^‡^	394 ± 138^¶^	446 ± 176^‡^	830 ± 438^§||^	1,035 ± 445*^†^
Glucose disposal t0–30	33 ± 17	35 ± 14^‡^	40 ± 14	44 ± 27^‡^	51 ± 22^§^	70 ± 23*^†^
Glucose disposal t60–210	73 ± 60^¶^	99 ± 81^‡^	239 ± 107^¶^	281 ± 162^‡^	611 ± 383^§||^	764 ± 405*^†^

Data are mean ± SD, *n* or *n*(%). *P* values are from ANOVA, *P* values between subgroups in post hoc Bonferroni analysis denoting statistical significance (*P* < 0.0125) are shown with symbols; * versus Cau NGT, ^†^versus Cau IFG/IGT, ^‡^versus Cau T2D, ^§^versus SA NGT, ^||^versus SA IFG/IGT, ^¶^versus SA T2D. ^A^newly identified individuals with T2D excluded, ^B^significance, ^C^non-significance with Student’s *t* test or *χ* ^2^ test *P* < 0.05, ^D^incomplete data, however, with a >75 % response rate

### Statistical analyses

We performed family-based analyses with the SOLAR software package [25]. Comparison between ethnicities was performed with variance component analyses adjusted for a number of covariates within SOLAR. For the prediction of NGT, IFG/IGT or T2D stage (WHO OGTT subgroup) in both ethnicities, we used ordinal regression analyses with SPSS version 15.0 for Windows (SPSS Inc., Chicago, IL, USA), adjusted for family ties, using a variable grouping each family with their own distinct number in SPSS. Data are expressed as mean ± SD, unless otherwise indicated. ANOVA were used for differences within given WHO OGTT subgroups and performed with SPSS; for each WHO OGTT subgroup, three comparisons were performed with ANOVA (unless otherwise stated); with the other two WHO OGTT subgroups of same ethnicity and with the corresponding other ethnic WHO OGTT subgroup. Inverse or log transformations were used when normality, or equal variance assumptions were not met. *P* value <0.05 was considered significant, unless otherwise stated.

## Results

In 36 South Asian families, 15 out of 37 (41 %) apparently healthy first-degree relatives were classified as IFG (*n* = 4), IGT (*n* = 5), the combination of IFG and IGT (*n* = 3) or newly diagnosed T2D (*n* = 3). In 24 Caucasian families, 13 (28 %) out of 47 apparently healthy first-degree relatives were classified as IFG (*n* = 3), IGT (*n* = 5), the combination of IFG and IGT (*n* = 4), while one was newly identified as T2D. For both ethnic groups, individuals with IFG and/or IGT were combined into one group of intermediate phenotypes and newly identified individuals with T2D were included with the original T2D cases in the diabetes group. The general characteristics of the three groups according to ethnicity are shown in Table 1. Waist circumference and W/H were lower in the groups with NGT compared with the other groups in both ethnicities. However, the relation of increasing W/H with glucose intolerance appeared to be less clear in South Asians when compared to Caucasians Notably, the South Asians with T2D were on average 10 years younger than the Caucasians with T2D and they already had a substantial prevalence of macrovascular disorders. Results from the OGTT demonstrated the following results; in both ethnicities with increasing glucose intolerance, glucose disposal increased, while both ISR t0–30 min and ISI decreased. Both ISR t0–210 min and ISR t60–210 min increased from NGT toward IFG/IGT, but decreased from IFG/IGT toward T2D. In general, ISR derived parameters in South Asians were markedly higher compared with the Caucasians, while ISI was lower with an overall between ethnicity difference of 3.8 ± 2.9 versus 6.5 ± 4.7, respectively (*P* < 0.001).

### Disposition indices, first/second phase beta-cell function

The unadjusted relationships between glucose disposal t0–210 min, ISR t0–30 min and ISR t60–210 min are shown in ternary plots (Supplementary Figure 1a, c). We determined the relationship between ISR t0–30 and ISR t60–210 with glucose disposal t0–210 using variance component analyses; after adjustment for ISI, age, W/H and gender the effect of early beta-cell function on glucose disposal t0–210 remained present in the South Asians, but disappeared in the Caucasian families, explaining the variance of glucose disposal t0–210 in our final model by 22.7 and 8.9 % in South Asian and Caucasian families, respectively (Table 2, Model 1). We also explored the effect of ISR t0–30 and ISI on ISR t60–210. The unadjusted relationships between ISI, ISR t0–30 and ISR t60–210 are shown in the ternary plots of Supplementary Figure 1b, d. After adjustment for age, W/H,WHO OGTT subgroup and gender, ISR t0–30 in South Asians had an effect on ISR t60–210, but such effects were not observed in Caucasian families, explaining 45.5 and 17.4 % of the variance of ISR t60–210 in our final model in South Asian and Caucasian families, respectively (Table 2, Model 2). We combined both ethnicities into an overall group and applied both Model 1 and 2 and tested for interaction between ethnicity and ISR t0–30 to glucose disposal t0–210 or ISR t60–210, respectively; only in Model 2, this interaction was significant (*β* = 0.341, [0.018;0.664]).

**Table 2:** SOLAR multiple regression analysis within family matrices

	**Model 1**	*β*	SE	Wald test	95 % CI	*P* value	**Model 2**	*β*	SE	Wald test	95 % CI	*P* value	**Model 3**	*β*	SE	Wald test	95 % CI	*P* value
SA	ISR t0–30	0.553	0.187	8.745	[0.186; 0.920]	**0.003**	ISRt0 − 30	0.707	0.212	11.122	[0.291; 1.123]	**<0.001**	DIratio	0.316	0.115	7.551	[0.091; 0.541]	**0.006**
	ISRt60–210	−0.211	0.197	1.147	[−0.597; 0.175]	0.284	ISI	−0.245	0.190	1.663	[−0.617; 0.127]	0.197	age	1.989	5.480	0.132	[−8.752; 12.730]	0.717
	ISI	−0.101	0.217	0.217	[−0.526; 0.324]	0.642	Age	−2.338	4.595	0.259	[−11.344; 6.668]	0.611	W/H	−0.001	0.001	2.778	[−0.002; 0.0001]	0.096
	age	−0.677	4.641	0.021	[−9,773; 8.419]	0.884	W/H	−0.0002	0.0002	1.000	[−0.0006; 0.0002]	0.317	WHO OGTT	2.456	2.240	1.202	[−1.934; 6.846]	0.273
	W/H	−0.00001	0.0002	0.003	[−0.0005; 0.0004]	0.956	WHO OGTT	0.402	1.872	0.046	[−3.267; 4.071]	0.830	Gender	12.557	115.011	0.012	[−212.865; 237.979]	0.913
	Gender	6.649	111.197	0.0036	[−211,297; 224,595]	0.952	Gender	−4.134	117.477	0.001	[−234.389; 226.121]	0.972						
	R^2^ (%)	22.7					R^2^(%)	45.5					R^2^ (%)	19.2				
Cau	ISRt0–30	0.092	0.178	0.267	[−0.257; 0.441]	0.605	ISRt0–30	0.270	0.156	2.996	[−0.035; 0.576]	0.083	DIratio	0.090	0.040	5.063	[0.012; 0.168]	**0.024**
	ISRt60–210	−0.057	0.188	0.092	[−0.425; 0.311]	0.762	ISI	−0.055	0.045	1.494	[−0.143; 0.033]	0.222	age	0.866	3.643	0.057	[−6.274; 8.006]	0.812
	ISI	−0.040	0.056	0.510	[−0.150; 0.070]	0.475	Age	−2.080	3.701	0.316	[−9.334; 5.173]	0.574	W/H	0.0002	0.0003	0.444	[−0.0004; 0.001]	0.505
	Age	2.909	4.182	0.484	[−5.288; 11.106]	0.487	W/H	0.0003	0.0003	1.000	[−0.0003; 0.001]	0.317	WHO OGTT	−0.030	0.030	1.000	[−0.089; 0.029]	0.317
	W/H	0.0003	0.0003	1.778	[−0.0002; 0.0008]	0.182	WHO OGTT	−0.020	0.030	0.444	[−0.079; 0.039]	0.505	Gender	**−**192.943	95.45	4.086	[−380.025; −5.861]	**0.043**
	Gender	−46.69	120.990	0.149	[−283.830; 190.450]	0.700	Gender	−125.358	93.631	1.793	[−308.975; 58.159]	0.181						
	R^2^ (%)	8.9					R^2^ (%)	17.4					R^2^ (%)	27.1				

Model 1 Trait: glucose disposal t0–210, Covariates ISR t030, ISR t60–210, ISI, Age, W/H, gender. Model 2 Trait ISR t60–210, Covariates ISR t0–30, ISI, age, W/H, WHO OGTT subgroup, gender. Model 3 Trait: glucose disposal t0–210, Covariates DI ratio, age, W/H, WHO OGTT subgroup, gender

Bold values indicate the significance of *P* values

**Fig. 1:**
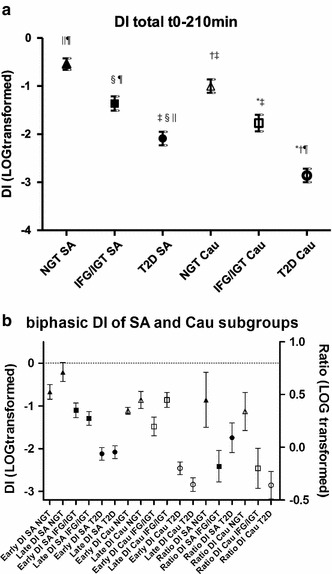
**a** Overall DI (DI t0–210) for all WHO OGTT subgroups (mean ± SEM) of both South Asian (*closed*) and Caucasian (*open*) families (*triangle* represents NGT, *square* IFG/IGT and *circle* T2D for both ethnicities). *P* values between subgroups in post hoc Bonferroni analysis denoting statistical significance (*P* < 0.0125) are shown with symbols; *versus Cau NGT, ^†^versus Cau IFG/IGT, ^‡^versus Cau T2D, ^§^versus SA NGT, ^||^versus SA IFG/IGT, ^¶^versus SA T2D. **b** Early DI (DI t0–30) and late DI (t60–210) on *left* *Y* axis in mean ± SEM, and ratio of late phase/early phase DI (*right* *Y* axis, mean ± SEM) for NGT, IFG/IGT and T2D of both South Asian (*closed*) and Caucasian (*open*) families (*triangle* represents NGT, square IFG/IGT and *circle* T2D for both ethnicities). South Asians: In early DI, there was a significant difference between NGT versus T2D and IFG/IGT versus T2D (*P* < 0.0001). In late DI, there was a significant difference between NGT versus IFG/IGT, NGT versus T2D and IFG/IGT versus T2D (*P* < 0.0001). In DI ratio, no significant differences were found (*P* = 0.14). Caucasians: In both early and late DI, there was a significant difference between NGT versus T2D and IFG/IGT versus T2D (both *P* < 0.0001, respectively). In DI ratio, there was a significant difference between NGT versus T2D (*P* = 0.016)

The overall DIs after logarithmic transformation of the three WHO OGTT subgroups according to ethnicity are shown in Fig. 1a. In both ethnicities, the overall DI decreased from the NGT to the IFG/IGT and further to the T2D groups. Illustrated in Fig. 1b, the early and late DIs of the South Asian WHO OGTT subgroups were higher than those of the equivalent Caucasian WHO OGTT subgroups, although a more rapid decline could be observed between South Asian NGT toward the IFG/IGT subgroup. In both ethnicities, early as well as late DI of both NGT and IFG/IGT subgroups differed significantly from their T2D subgroup. We examined the ratio of early and late phase DI in the WHO OGTT subgroups (Fig. 1b). In both the South Asian and the Caucasian families, the IFG/IGTgroup had lower ratios compared with the NGT group. This ratio was substantially higher in the South Asians with T2D compared with their relatives with IFG/IGT, whereas in the Caucasian families, a very low late phase DI resulted in the lowest ratio.

Adjusted for age, W/H, WHO OGTT subgroup and gender, DI ratio had an effect on glucose disposal t0–210 in both ethnicities, with the explained variances of glucose disposal t0–210 in our final model of 19.2 and 27.1 % in South Asian and Caucasian families, respectively (Table 2, Model 3). However, gender also played a role in Caucasian families, but not in South Asian families.

**Fig. 2:**
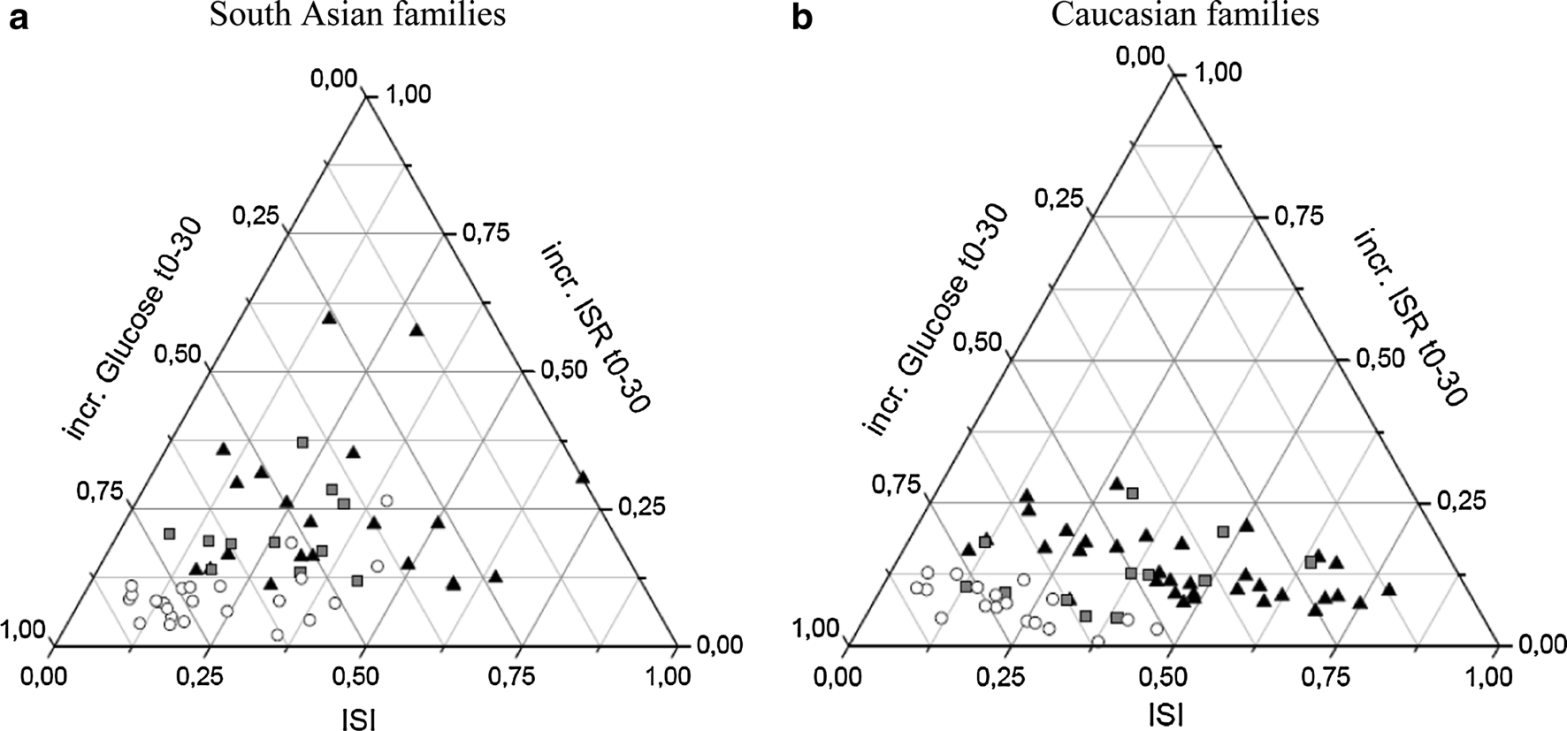
**a**, **b** Ternary plot of relationship between insulin sensitivity (ISI), early phase beta-cell function (ISR t0–30) and glucose disposal (incr. glucose AUC t0–30) based on OGTT from South Asian (*figures left*) and Caucasian families (*triangle* represents NGT, *square* IFG/IGT and *circle* T2D for both ethnicities)

Finally, to explore the differences in glucose handling in the WHO OGTT subgroups within the ethnicities, ternaries based on early DI are shown in Fig. 2a–b. (for total overview, three ternaries based on overall, early and late DI are shown in online Supplementary Fig. 2a–f). We used ordinal regression analyses to examine the nature of the components forming early and late DI. The ordinal analyses based on early DI parameters to predict WHO OGTT subgroups, adjusted for family ties, are given in Table 3. In contrast to Caucasians, there was an exclusive role for early beta-cell function, and not ISI, in predicting glucose tolerance in the South Asian families. Even when including early glucose disposal (glucose disposal t0–30) as an additional covariate in the analysis, ISR t0–30 remained the single significant predictor. In the Caucasian families, both ISR t0–30 and ISI contributed significantly. For both ethnicities, similar ordinal analyses based on late DI parameters did not show significant effects with the exception of late glucose disposal t60–210 (data not shown). Figure 2a–b suggest that the groups with T2D occupy a more distinct area toward the left corner of the ternaries, whereas the other two groups overlap more in the center. Therefore, we also performed logistic regression, adjusted for family ties, with the T2D groups versus the other relatives, the results can be found in Table 3; again, ISR t0–30 remained the most important discriminating variable in South Asians, even when glucose disposal t0–30 was included. For both ethnicities, similar binary logistic regression analyses based on late DI parameters demonstrated glucose disposal t60–210 as the most discriminating variable (data not shown).

**Table 3:** Ordinal and binary logistic regression analysis in both ethnicities predicting WHO OGTT subgroups adjusted for family ties

Independent	B	SE	Wald	95 % CI	*P* value
Ordinal regression analysis with NGT, IFG/IGT and T2D as dependent variables					
SA					
ISR t0–30	−0.020	0.009	5.246	[−0.037; −0.002]	**0.022**
ISI	−0.782	0.582	1.806	[−1.922; 0.359]	0.179
Cau					
ISR t0–30	−0.066	0.020	11.128	[−0.105; −0.027]	**0.001**
ISI	−0.603	0.256	5.556	[−1.105; −0.101]	**0.018**
Binary logistic regression analysis with T2D/non T2D as dependent variable					
SA					
ISR t0–30	−0.029	0.009	11.350	[−0.047; −0.011]	**0.001**
ISI	−0.338	0.275	1.516	[−0.877; 0.201]	0.218
Cau					
ISR t0–30	−0.017	0.006	7.859	[−0.029; −0.005]	**0.005**
ISI	−0.319	0.124	6.630	[−0.562; −0.076]	**0.01**

Bold values indicate the significance of *P* values

## Discussion

Across WHO OGTT subgroups from South Asian families, including the NGT group, we observed more insulin resistance, with more rapid decline of both early and late DI in NGT toward IFG/IGT, suggestive of early onset beta-cell failure. Across the WHO OGTT subgroups in Caucasian families, we observed a clear trend from normal insulin sensitivity to insulin resistance, while the DI decreased. Among the South Asians, the early insulin response explained at least partly the late insulin response as well as the overall glucose disposal. The ratio of the late over early DI decreased in both ethnicities from NTG to IFG/IGT, but waxed in the South Asian T2D and waned in the Caucasian T2D group, resulting in significant, but opposing effects in both ethnicities on the overall glucose disposal. The South Asians developed overt T2D at young age, while they still had a relatively high DI ratios. As a result of the lack of variance between the South Asian WHO OGTT subgroups in insulin sensitivity, only the early ISR predicted glucose tolerance state. Taken together, our findings confirm that changes in beta-cell dynamics play a prominent role in the development of T2D in South Asians. In Caucasians, more gradual processes of increasing resistance to insulin and decreasing overall insulin secretion seem to take place.

Our data confirm that—without adjustment for insulin sensitivity—South Asian individuals wrongly seem to have enough beta-cell capacity (Supplementary Table 1) with an above-average ability to secrete insulin [15]. Unfortunately, this compensatory beta-cell function is insufficient, leading to very early onset of T2D, as shown by the young age of manifest T2D in South Asians. These observations underline the important role of changes in beta-cell function, which have been reported to be the main contributor to abnormal glucose tolerance among a wide range of ethnicities, and are in line with increasing genetic evidence for beta-cell defects as an important predisposing factor for T2D [16, 17]. Hypersecretion of insulin may reflect beta-cell responses to different signals or a combination of an increased potentiating effect of glucose on beta-cells, long-lasting adaptation to severe insulin resistance and/or problems with the processing of insulin.

Clamp studies have demonstrated decreased insulin sensitivity among healthy South Asians when compared to other healthy controls [26–29]. We also found a decreased insulin sensitivity in the South Asians compared with the Caucasians. In contrast to the Caucasians, the degree of insulin sensitivity did not change between the three South Asian WHO OGTT subgroups. This very strong familial aggregation of insulin resistance suggests a strong contribution of environmental factors. However, we cannot infer from our data whether lifestyle, type of food, microbiome or other factors are involved. Among our families with high risk of T2D, the South Asians had much earlier onset of signs and symptoms of T2D compared with Caucasians. Notably, the burden from macrovascular disease was larger in our South Asian families, even in relatives who did not have T2D. This suggests that the severe insulin resistance of the South Asians contributes strongly to atherogenesis.

In addition to a demanding insulin resistant environment, failing beta-cell capacity is a major susceptibility factor to T2D in South Asian families, as was supported by recent genome wide association studies (GWAS). These studies show greater effects of SNP variants in beta-cell-related genes in South Asians than in other populations [30].

The strength of the present study is that it was a family-based approach and analysis of two ethnic groups, among various stages of glucose tolerance. Moreover, beta-cell and insulin sensitivity indices were based on multiple sampled prolonged OGTT’s. The relatively small numbers within the WHO OGTT subgroups of the families are a potential weakness, but a characteristic of both extensive phenotyping and family analyses is that it can be performed in relatively small populations. In line, we were able to observe beta-cell function alterations in a very consistent way.

## Conclusion

Based on extended OGTT measurements, we found that insulin sensitivity is already lower in South Asian than in Caucasian people with NGT. Insulin resistance in the South Asians does not change much during progression of glucose intolerance, and beta-cell dysfunction might play a dominant role in the early development of T2D among South Asian families in the Netherlands.

## Electronic supplementary material

Below is the link to the electronic supplementary material.

Supplementary material 1 (DOC 3044 kb)

## Abbreviations

CIR: Corrected insulin response
DI: Disposition index
HOMA-B: Homeostatic model assessment for beta-cell function
IGI: Insulinogenic index
ISEC: Insulin SECretion
ISI: Insulin sensitivity index
ISR: Insulin secretion rate
MRCi: Metabolic clearance rate of insulin
