# Supplementary material for: Failing beta-cell adaptation in South Asian families with a high risk of type 2 diabetes
Source: Acta Diabetol. 2014 May 5;52(1):11–9. doi: 10.1007/s00592-014-0588-9 (PMC4340485; doi:10.1007/s00592-014-0588-9)
Supplement: Supplementary file 1 — Supplementary material 1 (DOC 3044 kb) [file 592_2014_588_MOESM1_ESM.doc]

**Failing beta-cell adaptation in South Asian families with a high risk of type 2 diabetes- Online Supplemental**

Short title: Beta cell dynamics in South Asian families

Sjaam Jainandunsing, MD^1^, Behiye Özcan, MD^1^, Trinet Rietveld^1^, Joram N.I. van Miert^1^, Aaron Isaacs, PhD^2^,Janneke G. Langendonk,MD, PhD^1^, Felix W.M. de Rooij, PhD^1^, Eric J.G. Sijbrands, MD, PhD^1^

^1^Department of Internal Medicine, Erasmus MC - University Medical Center Rotterdam,The Netherlands.

^2^ Department of Epidemiology, Erasmus MC - University Medical Center Rotterdam,The Netherlands.

**Address all correspondence and reprint requests to:**

F.W.M. de Rooij

Department of Internal Medicine

Erasmus MC room Bd-299

PO-box 2040

3000 CA Rotterdam, The Netherlands

Tel: (+31) 1070 35457

Fax: (+31) 10 70 33964

E-mail: f.derooij@erasmusmc.nl

***Supplementary Table 1*** *Measures from the OGTT in persons with NGT, IGT and/or IFG and T2D*

|  | **South Asian families** | | | **Caucasian families** | | |
| --- | --- | --- | --- | --- | --- | --- |
|  | **NGT** | **IFG/IGT** | **T2D** | **NGT** | **IFG/IGT** | **T2D** |
| *n* | 22 | 12 | 23 | 34 | 12 | 18 |
| $I_{0}/G_{0}$*(pmol/mmol)* | 12.5±1.7 | 12.0±2.5 | 13.2±3.1 | 8.4±1.1 | 8.7±1.4 | 9.5±2.0 |
| $Cpep_{0}/G_{0}$*(pmol/mmol)* | 143.8±12.2 | 157.8±20.0 | 129.2±7.4 | 106.3±6.5 | 114.2±10.5 | 120.4±16.7 |
| $I_{30}/G_{30}$*(pmol/mmol)* | 86.0±12.5*¶ | 64.0±13.5 | 30.4±4.7§ | 43.9±4.7‡§ | 33.5±5.5 | 19.8±3.3* |
| $Cpep_{30}/G_{30}$*(pmol/mmol)* | 467.0±38.4*¶ | 351.2±36.1(25%↓)¶ | 196.3±20.6§|| | 327.0±21.0‡§ | 243.8±23.3 | 157.1±18.7* |
| $HOMA-B$ | 109.5±15.4 | 86.8±20.2 | 80.7±22.2 | 73.4±9.7 | 59.3±8.6 | 50.7±11.0 |
| $\Delta I_{0-30}/\Delta G_{0-30}$*(pmol/mmol)* | 177.7±93.9 | 168.0±40.3 | 69.5±15.4 | 144.8±23.8‡ | 89.0±20.7 | 41.2±8.1* |
| $\Delta Cpep_{0-30}/\Delta G_{0-30}$*(pmol/mmol)* | 978.4±368.6 | 764.3±115.1 | 352.4±77.9 | 1043.0±162.7‡ | 569.3±111.8 | 235.9±29.1* |
| $\Delta I_{0-30}/G_{30}$*(pmol/mmol)* | 76.9±11.9*¶ | 56.1±13.5 | 21.7±3.8§ | 38.3±4.4‡§ | 27.7±5.5 | 13.8±2.5* |
| $\Delta Cpep_{0-30}/G_{30}$*(pmol/mmol)* | 364.1±32.3*¶ | 246.8±34.2¶ | 112.6±19.0§|| | 252.9±18.2‡§ | 168.1±21.4 | 81.6±10.5* |
| $CIR$ | 2646.1±470.5*¶ | 1301.2±298.8 | 538.9±136.0§ | 1254.3±142.4‡§ | 676.0±129.4 | 252.1±44.7* |
| $incrAUC_{ins0-30}/incrAUC_{gluc0-30}$*(pmol/mmol)* | 298.0±87.9*¶ | 172.0±32.9 | 66.3±14.9§ | 117.6±11.8‡§ | 90.8±18.8 | 47.4±7.8* |
| $incrAUC_{Cpep0-30}/incrAUC_{gluc0-30}$*(pmol/mmol)* | 1654.2±476.6¶ | 812.4±114.7 | 351.3±83.4§ | 817.0±70.3‡ | 582.7±114.4 | 224.4±25.6* |
| $incrAUC_{ins0-120}/incrAUC_{gluc0-120}$*(pmol/mmol)* | 587.3±129.3*¶ | 224.3±26.7 | 111.3±28.4§ | 268.0±41.0‡§ | 124.7±20.9 | 48.2±8.6* |
| $incrAUC_{Cpep0-120}/incrAUC_{gluc0-120}$*(pmol/mmol)* | 2859.8±468.2||¶ | 1051.6±131.6§ | 580.9±131.2§ | 2075.7±339.1‡ | 794.6±117.3 | 271.1±40.3* |
| $incrAUC_{ins120-210}/incrAUC_{gluc120-210}$*(pmol/mmol)* | 1971.0±615.2¶ | 527.9±83.7 | 265.2±77.0§ | 1161.5±665.8 | 847.1±514.1 | 128.8±37.0 |
| $incrAUC_{Cpep120-210}/incrAUC_{gluc120-210}$*(pmol/mmol)* | 12601.8±3049.7¶ | 2754.7±306.8 | 1882.4±477.1§ | 7484.9±2588.1 | 5917.7±3193.7 | 889.8±174.2 |
| $1^{st}phase Stumvoll$*(pmol/l)* | 1801.6±171.4¶ | 1362.2±158.8 | 724.1±177.5§ | 1242.5±110.8‡ | 1061.5±182.2‡ | 252.2±157.8*† |
| $2^{d}phase Stumvoll$*(pmol/l)* | 464.1±43.0¶ | 364.7±39.5 | 218.9±42.1§ | 328.4±27.5‡ | 287.9±43.4 | 108.5±41.5* |
| $MCRi$ (l/min) | 1.8±0.2* | 1.6±0.2 | 2.0±0.1 | 2.5±0.2§ | 2.4±0.2 | 2.8±0.4 |

*Data are means+/- SEM. P values between subgroups in ANOVA post-hoc Bonferroni analysis denoting statistical significance (P<0.0125) are shown with symbols; ;* *=versus Cau NGT, † =versus Cau IFG/IGT, ‡ = versus Cau T2D, § = versus SA NGT, || = versus SA IFG/IGT, ¶ = versus SA T2D

Indices of Beta-cell function

We calculated the fasting insulin/glucose ratio ($I_{0}/G_{0}$), fasting C-peptide/glucose ratio ($Cpep_{0}/G_{0}$) and homeostatic model assessment for beta-cell function (HOMA-B) using

Early insulin response indices in OGTT were the insulin/glucose ratio at t=30min ($I_{30}/G_{30}$), C-peptide/glucose ratio at t=30min ($Cpep_{30}/G_{30}$);the insulinogenic indices (IGI) for the increase in insulin and C-peptide as response to the increment in glucose within first 30 min after the glucose-load:: $\Delta I_{0-30}/\Delta G_{0-30}$, $\Delta Cpep_{0-30}/\Delta G_{0-30}$, $\Delta Cpep_{0-30}/G_{30}$ and $\Delta I_{0-30}/G_{30}$the corrected insulin response (CIR) was calculated as and the incremental area under curve (AUC) ofinsulin and C-peptide concentrations in response to the increment in glucose during the OGTT as: $incrAUC_{ins0-30}/incrAUC_{gluc0-30}$ and$incrAUC_{Cpep0-30}/incrAUC_{gluc0-30}$

Late insulin response indices in OGTT were calculated as:$incrAUC_{ins120-210}/incrAUC_{gluc120-210}$, $incrAUC_{Cpep120-210}/incrAUC_{gluc120-210}$, and overall insulin response indices as$incrAUC_{ins0-120}/incrAUC_{gluc0-120}$ and$incrAUC_{Cpep0-120}/incrAUC_{gluc0-120}$

Estimates of beta-cell function according to Stumvoll, et al. for first phase and second phase insulin release among NGT and IGT individuals were calculated with the following formulas: $1^{st}phase=728+3.537\times Ins_{0}-120.3\times Gluc_{60}+1.341\times Ins_{60}+21.27\times BMI$,

$2^{d}phase=208+0.335\times Ins_{60}-26.33\times Gluc_{60}+0.887\times Ins_{0}+3.933\times BMI$.

Metabolic clearance rate

The metabolic clearance rate of insulin (MCri) was calculated from the ratio between the total AUC of the ISR and the AUC of total insulin measured in plasma: $AUC_{ISR0-210}/AUC_{ins0-210}$

**Legends to Figures;**

*Supplementary figure 1*: Ternary plot of ISR t0-30 and ISR t60-210 with glucose disposal (left) or ISI (right) in South Asian (top panel) and Caucasian(below) families(triangle NGT, square IFG/IGT, circle T2D).

*Supplementary figure 2:* Ternary plot of ISI, ISR t0-210 and glucose disposal t0-210 based on OGTT in South Asian and Caucasian families (left side and right side respectively). Also, ternary plots of relationship between ISI, ISR t0-30 and glucose disposal t0-30 are depicted in the middle and ternary plots of ISI, ISR t60-210 and glucose disposal t60-210 are depicted below ( triangle NGT, square IFG/IGT, circle T2D)

**Supplementary figure 1**

**South Asian families**


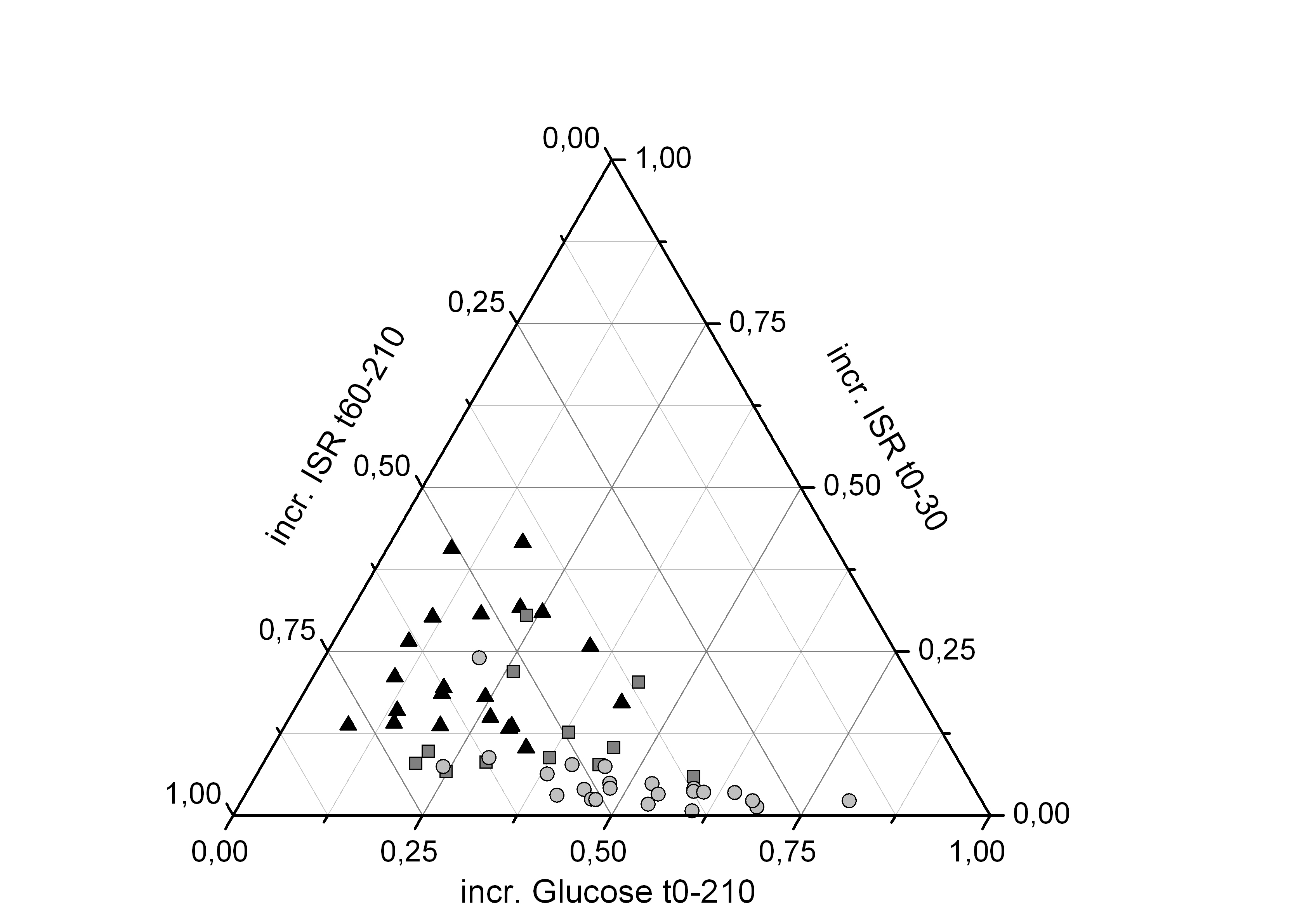

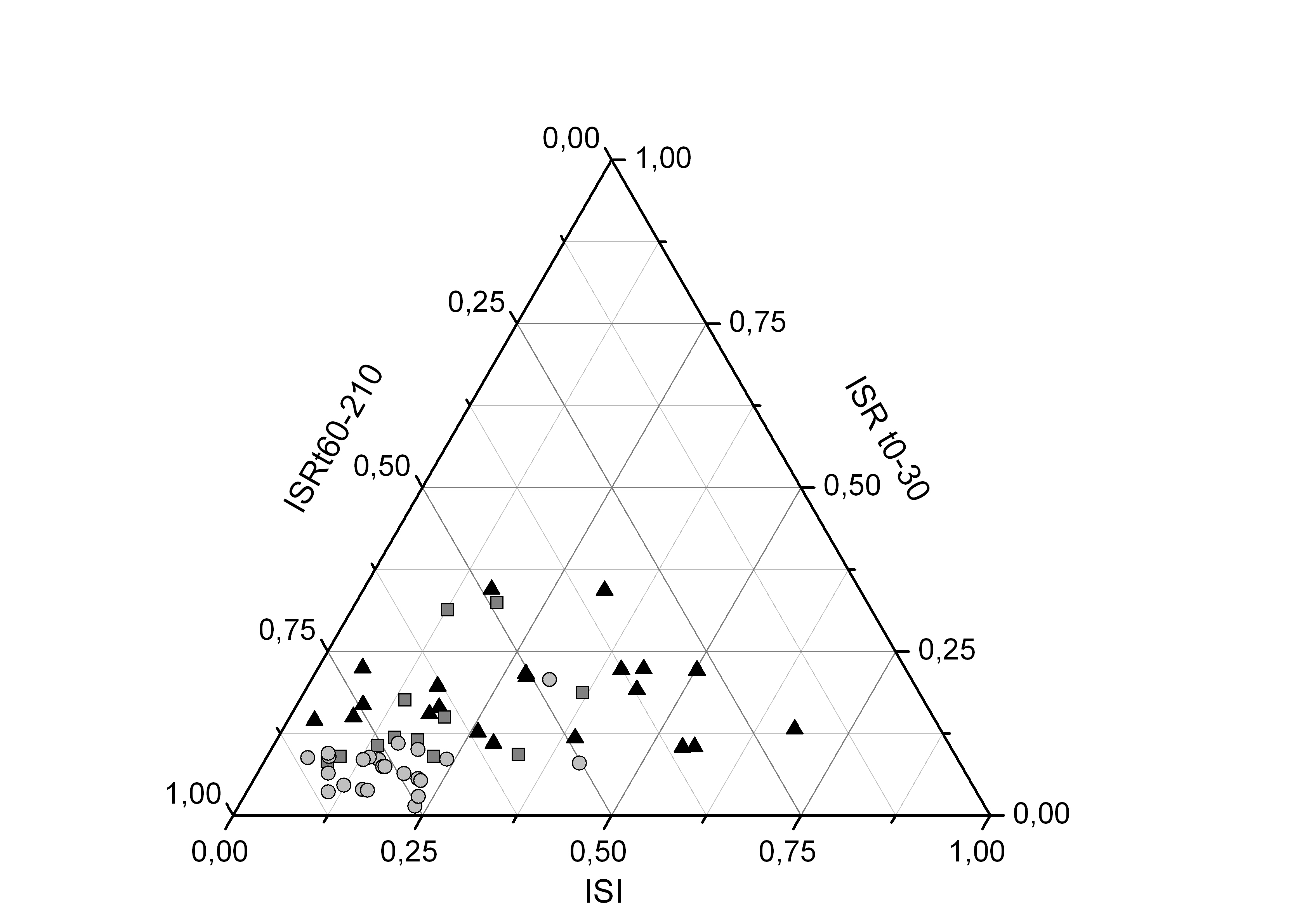


**A B**

**Caucasian families**


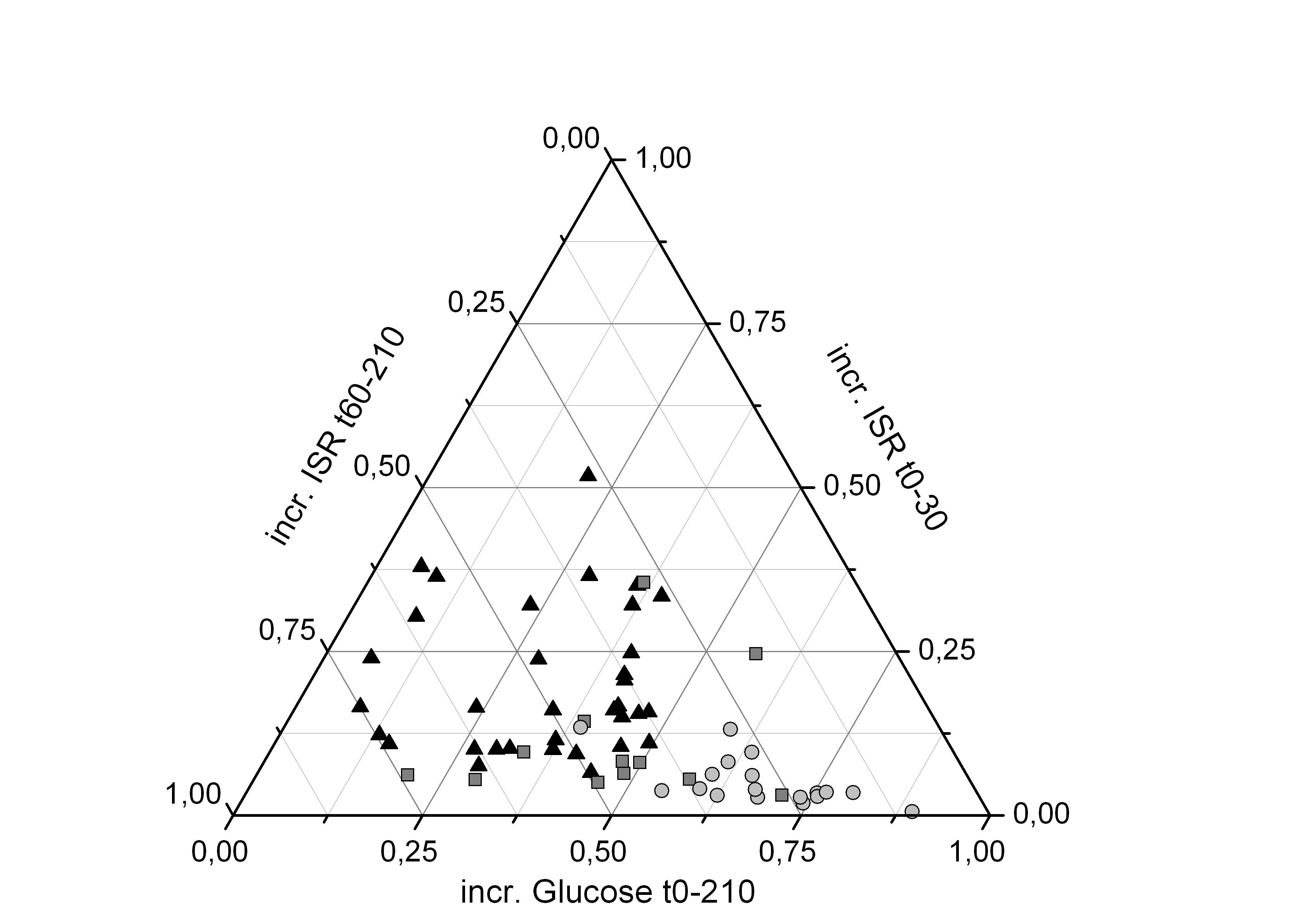

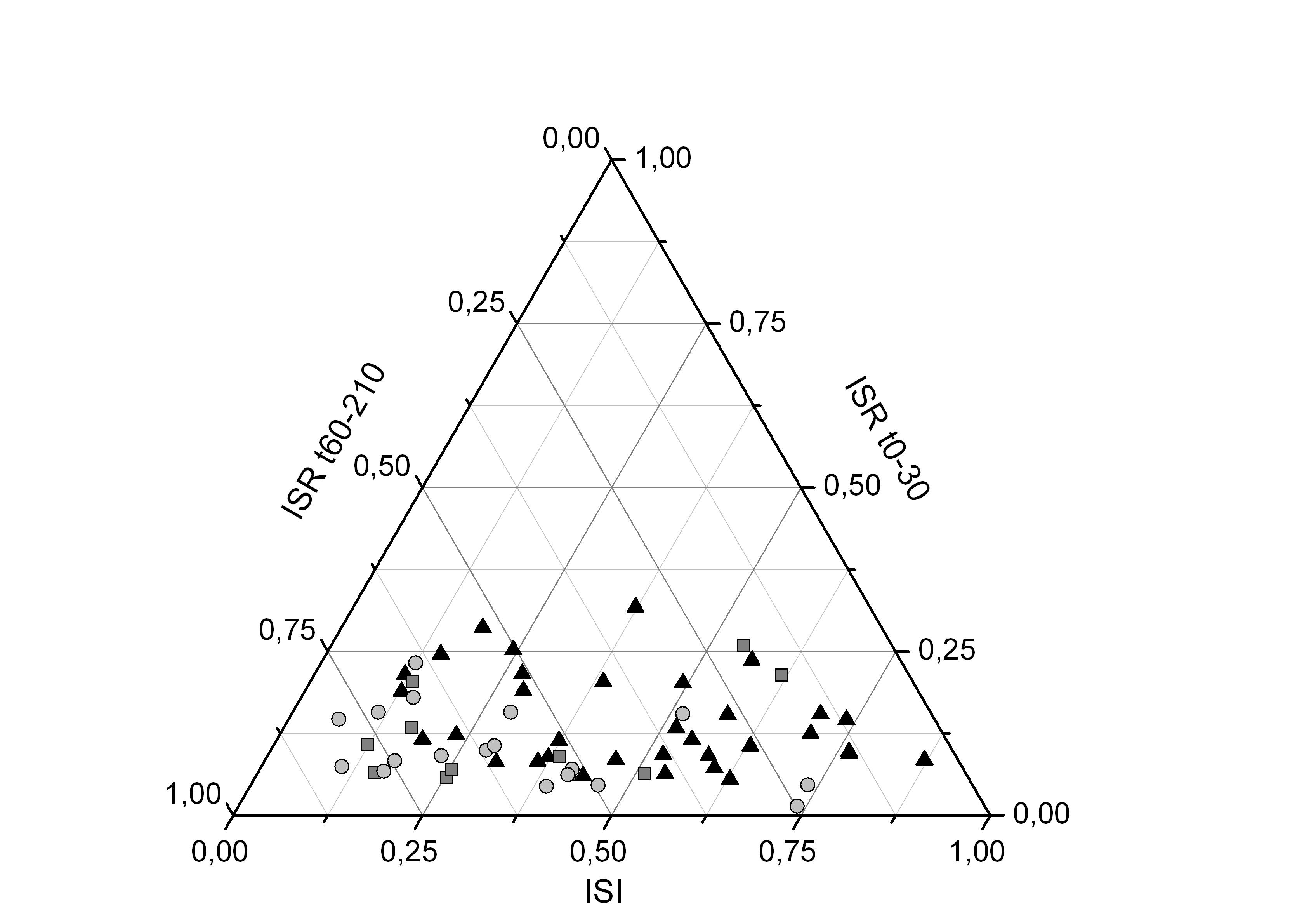


**C D**

**Supplementary figure 2**

South Asian families Caucasian families

A B

C D

E F

1. Matthews, D.R., et al., *Homeostasis model assessment: insulin resistance and beta-cell function from fasting plasma glucose and insulin concentrations in man.* Diabetologia, 1985. **28**(7): p. 412-9.

2. Seltzer, H.S., et al., *Insulin secretion in response to glycemic stimulus: relation of delayed initial release to carbohydrate intolerance in mild diabetes mellitus.* J Clin Invest, 1967. **46**(3): p. 323-35.

3. Guerrero-Romero, F. and M. Rodriguez-Moran, *Glucose intolerance is predicted by the high Fasting Insulin-to-Glucose ratio.* Diabetes Metab, 2001. **27**(2 Pt 1): p. 117-21.

4. Wareham, N.J., et al., *The 30 minute insulin incremental response in an oral glucose tolerance test as a measure of insulin secretion.* Diabet Med, 1995. **12**(10): p. 931.

5. Sluiter, W.J., et al., *Glucose tolerance and insulin release, a mathematical approach I. Assay of the beta-cell response after oral glucose loading.* Diabetes, 1976. **25**(4): p. 241-4.

6. Utzschneider, K.M., et al., *Within-subject variability of measures of beta cell function derived from a 2 h OGTT: implications for research studies.* Diabetologia, 2007. **50**(12): p. 2516-25.

7. Stumvoll, M., et al., *Oral glucose tolerance test indexes for insulin sensitivity and secretion based on various availabilities of sampling times.* Diabetes Care, 2001. **24**(4): p. 796-7.

8. Bendsen, N.T., et al., *Effect of trans-fatty acid intake on insulin sensitivity and intramuscular lipids--a randomized trial in overweight postmenopausal women.* Metabolism. **60**(7): p. 906-13.
